# Fractional-Order PID Control Strategy on Hydraulic-Loading System of Typical Electromechanical Platform

**DOI:** 10.3390/s18093024

**Published:** 2018-09-10

**Authors:** Ning Wang, Jianmei Wang, Zhixiong Li, Xuefeng Tang, Dingbang Hou

**Affiliations:** 1 Coordinative Innovation Center of Taiyuan Heavy Machinery Equipment, Taiyuan University of Science and Technology, Taiyuan 030024, China; wnwywhf553@163.com (N.W.); m15135167380_1@163.com (X.T.); 13623664261@163.com (D.H.); 2 School of Mechanical, Materials, Mechatronic and Biomedical Engineering, University of Wollongong, Wollongong, NSW 2522, Australia; zhixiong.li@cumt.edu.cn; 3 School of Mechatronics Engineering, China University of Mining and Technology, Xuzhou 221116, China

**Keywords:** fractional-order PID controller, electro-hydraulic system, system identification, journal bearing test rig

## Abstract

In this paper, a control method for a hydraulic loading system of an electromechanical platform based on a fractional-order PID (Proportion-Integration-Differentiation) controller is proposed, which is used to drive the loading system of a mechatronic journal test rig. The mathematical model of the control system is established according to the principle of the electro-hydraulic system. Considering the indetermination of model parameters, the method of parameter identification was used to verify the rationality of the theoretical model. In order to improve the control precision of the hydraulic loading system, the traditional PID controller and fractional-order PID controller are designed by selecting appropriate tuning parameters. Their control performances are analyzed in frequency domain and time domain, respectively. The results show that the fractional-order PID controller has better control effect. By observing the actual control effect of the fractional-order PID controller on the journal test rig, the effectiveness of this control algorithm is verified.

## 1. Introduction

A journal bearing test rig as a typical electromechanical platform was developed by authors to study the integrated performance of journal bearing under various load rolling. The test rig uses the hydraulic cylinder to apply loads to test bearing. The hydraulic system uses the hydraulic pump as the pressure oil source and adjusts the loads through the electro-hydraulic proportional relief valve. The loads are set by the monitoring interface of an industrial personal computer. Real-time loads are collected through pressure sensors. In order to simulate the fluctuating load under different rolling conditions and different rolling processes, the reliability of the experimental data and the control precision must be guaranteed.

A hydraulic system is a typical nonlinear system. Some of the parameters, such as flow coefficient, oil viscosity and elastic modulus, are uncertain. The traditional PID (Proportion-Integration-Differentiation) controller is widely used in the electro-hydraulic system, which is simple and easy to implement. However, due to the nonlinear factors of the hydraulic system, it is difficult to achieve satisfactory results. Several advanced control methods are put forward for the hydraulic system. Karam and Elbayomy et al. proposed the PID controller based on a genetic algorithm to control the electro-hydraulic servo system, and proved its effectiveness through experiments [1]. An adaptive sliding control method is presented for an electro-hydraulic system with nonlinear unknown parameters by Guan and Pan [2]. The adaptive robust control is commonly used in the hydraulic system. It uses a fixed controller to satisfy the control quality with uncertain objects [3,4,5]. Nonlinear robust control methods are used for the hydraulic load simulator, which achieve a guaranteed transient performance and final tracking accuracy in the presence of both parametric uncertainties and uncertain nonlinearities [6,7]. A grey prediction model combined with a fuzzy PID controller is designed to improve the control quality of the loading system while eliminating or reducing the disturbance [8]. In the literature [9,10], the nonlinear version of quantitative feedback theory (QFT) is employed to design a robust time-invariant controller for a hydraulic actuator. Truong and Ahn use the proportional integral derivative (PID) control method, as well as an online self-tuning fuzzy-neural mechanism to improve the control performance of the loading system [11]. Yao et al. developed and applied a neural network adaptive inverse controller to an electro-hydraulic servo system which is capable of tracking desired signals with high accuracy, and has good real-time performance [12]. Milić et al. discuss the use of the techniques based on linear matrix inequalities for robust H ∞ position control synthesis of an electro-hydraulic servo system [13]. Feed-forward inverse model (FFIM) control is also used in the electro-hydraulic hybrid system to improve the robustness of the system [14,15,16]. These controllers all have their own advantages and limitations. The disadvantage of the adaptive sliding control method is that it will produce high jitter, which may activate the unmodeled high jitter components in the system, and even make the system unstable. The design of a robust control system is usually completed by senior experts. Once the controller is designed, its parameters may not be easy to change. The disadvantage of the nonlinear version of quantitative feedback theory is that a lot of quantitative calculation and analysis is needed. The neural network algorithm has the disadvantage of slow learning speed and easily falls into local extreme value. The design of the fuzzy controller relies on engineering experience and lacks systematic theoretical basis. The feed-forward inverse model (FFIM) control method presents a low computational burden, particularly compared to the standard model predictive control algorithms. These usually require considerable computational effort, which often thwarts their implementation on real industrial systems. In recent years, more and more researchers have been concentrated on the fractional order PID (FOPID) controller. The first report of a fractional PID controller was published by Podlubny in 1994 [17]. The TID (Tilted Proportional and Integral) controller, CRONE controller, *PI^λ^D^μ^* controller, and fractional lead-lag compensator were introduced briefly by Xue and Chen in 2002 [18]. A method for stabilizing fractional PID controllers for time delay systems was proposed by Hamamci [19,20]. The synthesis of FOPID controllers regarding stability using Hermite Biehler theorem was presented by Caponetto et al. [21]. Internal Model Control (IMC) based tuning was proposed by Tavakoli-Kakhki and Haeri in 2010 [22]. A fractional PID controller for nonlinear systems was designed by Barbosa et al. in 2007 [23]. An adaptive sliding mode (ASMC) was proposed for the fractional-order chaotic system by Yin et al. in 2013 [24]. A neural network-based design for a fractional PID controller was presented [25]. Because of the five parameters of the FOPID controller, parameter tuning becomes difficult. Various tuning methods for controllers of integer/fractional order are proposed. Radac et al. present a new iterative data-driven algorithm (IDDA) for the experiment-based tuning of controllers for nonlinear systems. This solves the optimization problems for nonlinear processes while using linear controllers accounting for operational constraints and employing a quadratic penalty function approach [26]. Sayyaf, Negin and Tavazoei present an analytical method to tune a fixed-structure fractional-order compensator for satisfying desired phase and gain margins with adjustable crossover frequencies [27]. In the literature [28], the adaptive neural control of a class of uncertain multi-input multi-output (MIMO) nonlinear time-delay non-integer order systems with unmeasured states, unknown control direction, and unknown asymmetric saturation actuator is introduced. Valerio and Costa [29] proposed tuning of the FPID controller based on Ziegler Nichols-type rules. An analytical tuning method for the fractional order system was proposed by Zhao et al. [30]. Over the past few decades, the FOPID controllers have gradually entered the field of industrial control, which have been applied in various aspects. In the literature [31], PID and FOPID controllers are applied to a four-pool irrigation canal extant. Muresan et al. propose a simple approach for designing a FOPI controller for controlling the speed of a DC motor [32]. Further, *PI^λ^D^μ^* controllers are designed for the automatic voltage regulator (AVR) system [33,34,35] and load frequency control (LFC) [36,37]. In the field of electro-hydraulic control, the FOPID controller has been gradually adopted by researchers. In the literature [38], the FOPID controller is adopted to control the electro-hydraulic system in an insulator fatigue test device. The results have shown that it is effective.

At present, research on the FOPID controller in the loading system of a bearing test rig is limited. The control performance of FOPID controller is studied in this paper, based on the load simulator of the existing test device. The mathematical model of the test rig control system is obtained using hydraulic theory. Since there are many uncertain parameters in the transfer function, it was decided that the response data with the MATLAB system identification toolbox would be analyzed to obtain the approximate transfer function of the hydraulic loading system. In order to verify the performance of the FOPID controller in the hydraulic loading system, the FOPID controller and traditional PID controller were both designed for the electro-hydraulic proportional control system. The control effect of FOPID and traditional PID are compared in time domain and frequency domain through simulation. Simulation results show the superiority of the FOPID controller. The experiment was carried out on the journal bearing test rig to verify the actual performance of the FOPID controller. The results show that the FOPID controller can improve the performance of the hydraulic loading system.

## 2. Hydraulic Loading System

### 2.1. Working Principles of System

**Figure 1:**
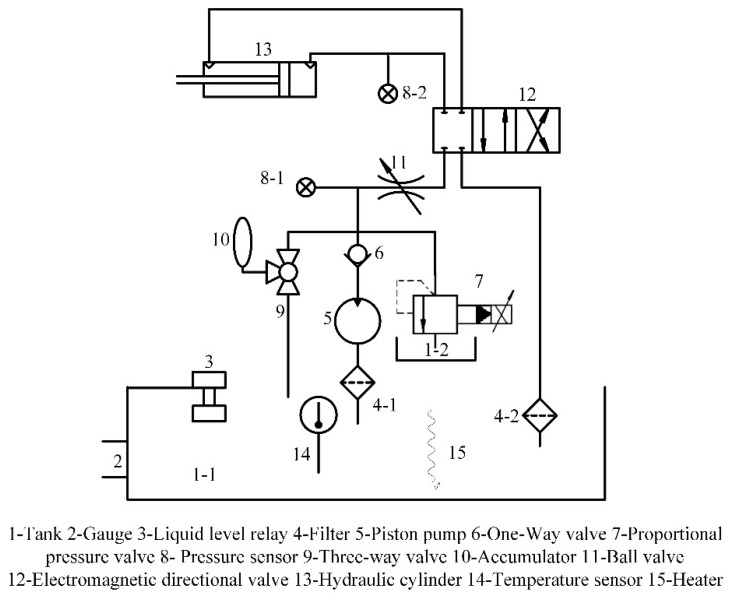
Principle diagram of hydraulic system.

The hydraulic loading system is used to simulate rolling force on the test bearing. The pressure range is 0–20 MPa and it is equivalent to 0–90 tons load. This system consists of a fuel tank, motor, axial piston pump, proportional valve, accumulator, cooler, digital display temperature controller, valve group (electromagnetic directional valve, direct-acting relief valve), liquid level relay, oil filter, and so forth). The specific hydraulic principle is shown in Figure 1.

**Figure 2:**
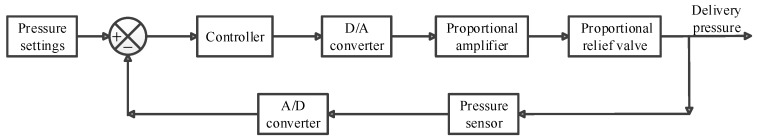
Control schematic diagram.

The control principle is shown in Figure 2.

The test rig uses the hydraulic cylinder to load the test bearing. When loading, the hydraulic cylinder contacts with the pressure sharing block on the bearing house. The hydraulic system adjusts the load by the electro-hydraulic proportional relief valve. The load value is given by the upper computer. The analog output module of the PLC outputs control current. The proportional amplifier amplifies the control current to drive the open degree of the electric-hydraulic proportional relief valve. Real-time load is collected through pressure sensors. The core components of the whole hydraulic loading system include a controller, proportional relief valve and pressure sensor, which jointly complete the pressure setting, control and detection of the system.

### 2.2. Model of Hydraulic Loading System

**Figure 3:**
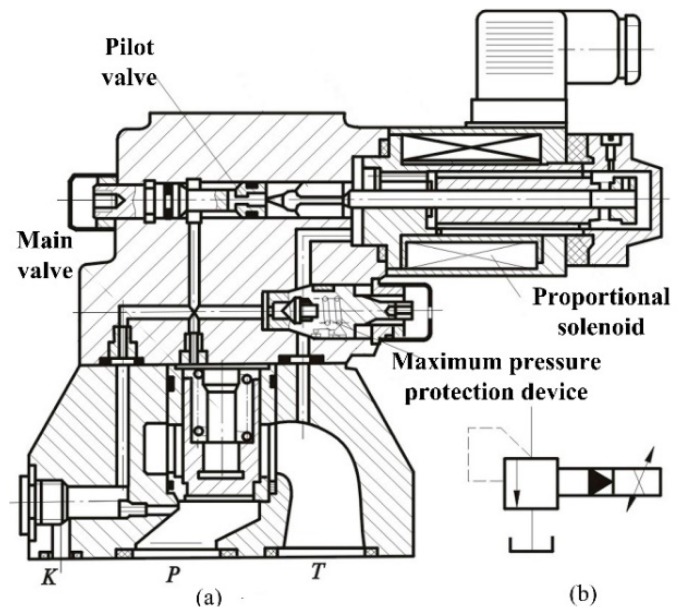
Electric-hydraulic proportional relief valve.

In order to facilitate the simulation and application of the control algorithm, the mathematical model of the current system needs to be established. The test rig adopts DBEM 10–30 b/200 YM type valve as a pilot relief valve. It is the core component that transforms the system pressure from the electrical signal to the actual pressure. The concrete structure is shown in Figure 3.

The valve is used for constant pressure. When the control signal is certain, stable system pressure can be obtained. The change of control signal can adjust the pressure of the system smoothly, so that the hydraulic impact on the system is small.

**Figure 4:**
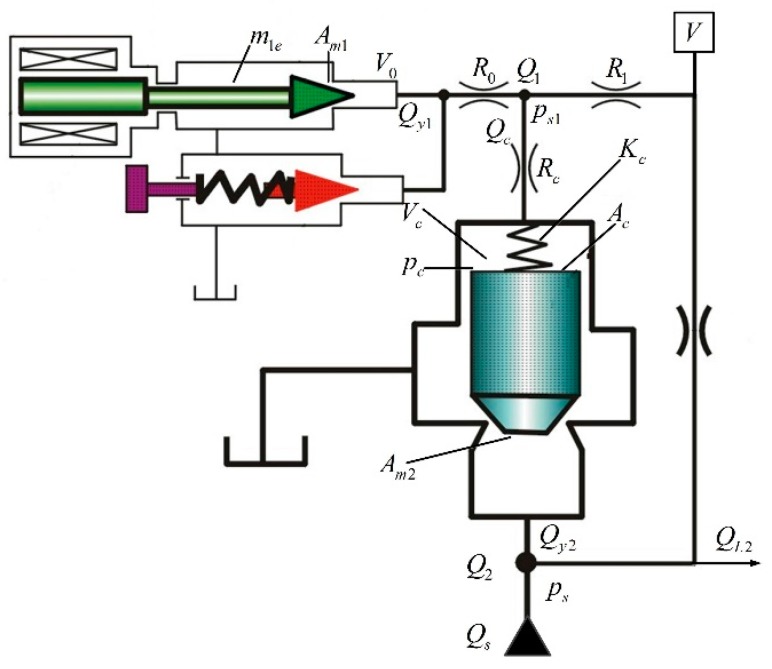
Working principle diagram.

The working principle of the electro-hydraulic proportional relief valve is shown in Figure 4. It includes a pilot valve and a main valve. The high-pressure fluid acts simultaneously on both ends of the main valve, so the force balance is maintained because of the same effective area on the main valve. If the pressure continues to increase, the force balance will be broken. When the hydraulic pressure reaches the thrust of the proportional electromagnet, the pilot conical valve opens. The fluid pressure will be fed through the pilot valve core. Pressure drop will be produced on the upper end of the main valve. The main valve core overcomes the rise of the spring force, and the P and T oil roads will be connected. The excess flow of the system flows back to the tank, so the pressure will not continue to rise and the reconstruction force will be balanced. Meanwhile, this proportional relief valve is equipped with a pressure relief valve. When the electrical or hydraulic system fails (due to excessive current, or excessive pressure in the hydraulic system), the safety device acts to limit the rise of system pressure. The parameters are listed in Table 1.

The mathematical transfer function model of the control system has been established [39]. In order to simplify the process of modeling, some assumptions are proposed because of some parameters of the model with small effects.

(1). The flow generated by the main valve movement is minimal compared to the flow of the valve port. Therefore, its channel is negligible.
(2). The flow of orifice $R_{1}$ is far less than the valve port flow, so its channel can be ignored.
(3). Compared with the turning frequency formed in the closed volume *V* of the pump outlet and the natural frequency of the main valve, the turning frequency of the proportional electromagnet, the natural frequency of the pilot valve, and the turning frequency of the B half-bridge are larger. Therefore, the proportional amplifier and proportional electromagnet can be considered as a proportional component.
(4). In order to simplify the model, the tiny disturbing force of the main valve spool is negligible.
(5). The pilot valve is controlled by electric signal. *V*1 and *Vc* are very small. Therefore, the integral effect of the two links can be omitted.

As shown in Figure 2, the establishment of a mathematical model should be carried out from the following parts.

(1). The proportional amplifier can handle the input signal according to the actual needs, which belongs to the driving device. It is considered as a proportional component and its coefficient of proportionality is *Ka*.
(2). The proportional electromagnet has a high response frequency compared to the hydraulic system. Therefore, the proportional electromagnet is also regarded as a proportional component. Its gain is *Ki*.
(3). The simplified transfer function of the pilot valve is: $\frac{P_{s1}(s)}{F_{e}(s)}=\frac{K_{q1}}{m_{1e}s^{2}+(B_{1e}+R_{0}A_{m1}^{2})s+K_{1e}}\cdot \frac{-1}{\frac{V}{E}s+C_{L1}}$.
(4). $P_{s1}(s)$ is an instruction signal for the output of the pilot valve. $F_{e}(s)$ is the driving force of the output of the proportional electromagnet.
(5). The main valve is the core part of the whole system. According to hydraulic related knowledge, the following equation can be obtained from the flow equation, continuity equation and force balance equation of the hydraulic system.

Equation (1) is the flow continuity equation of the cavity $Q_{1}$ in a pilot liquid bridge;

(1) $$Q_{R1}(s)=Q_{y1}(s)+\frac{V_{1}}{E}sp_{s1}(s)+Q_{c}(s)$$

$Q_{y1}(s)$ is the flow to volume $V_{0}$, and $Q_{R1}(s)$ is the flow of $Q_{1}$. Equation (2) was obtained from the throttle effect of liquid resistance $R_{1}$;

(2) $$p_{s}(s)-p_{s1}(s)=R_{1}Q_{R1}(s)$$

Equation (3) is derived from the throttle effect of liquid resistance $R_{c}$;

(3) $$P_{s1}(s)-P_{c}(s)=R_{c}Q_{c}(s)$$

In Equation (3), $Q_{c}(s)$ is the flow from the control chamber $V_{c}$ passing in and out the B half-bridge;

Flow continuity Equation (4) in cavity $V_{c}$;

(4) $$Q_{c}(s)=\frac{V_{c}}{E}sp_{c}(s)+A_{c}sx_{v2}(s)$$

Flow continuity equation for the inlet cavity $Q_{2}$ of the main valve.

(5) $$Q_{s}(s)-Q_{L2}(s)=\frac{V}{E}sp_{s}(s)+A_{m2}sx_{V2}(s)+Q_{R1}(s)+Q_{y2}(s)+C_{L}p_{s}(s)$$

Force balance equation on the main valve spool.

(6) $$A_{c}p_{c}(s)-A_{m2}p_{s}(s)-F_{L}(s)=(m_{2}s^{2}+B_{2}s+K_{s2})x_{v2}(s)$$

Pressure-flow equation of main valve port.

(7) $$Q_{y2}(s)=K_{q2}x_{V2}(s)-K_{c2}p(s)$$

$Q_{c}(s)$ is the load flow of the pilot relief valve, therefore $Q_{c}(s)=Q_{L1}(s)$. Some parameters in the equation are shown in Table 2.

The pressure sensor is also considered as a proportional component, and its sensor coefficient is set to $K_{f}$.

**Figure 5:**
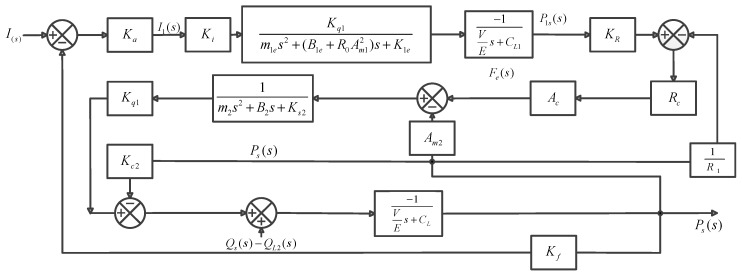
Transfer function block diagram.

The following transfer function can be obtained according to Figure 5.

(8) $$G(s)=\frac{\frac{K_{a}K_{i}K_{q1}K_{R}\frac{A_{c}R_{c}K_{q2}K_{q1}}{m_{1e}s+(B_{1e}+RA_{m1}^{2})s+K_{1e}}}{m_{2}s^{2}+B_{2}s+K_{s2}}\cdot \frac{-1}{\frac{V}{E}s+C_{L1}}-Q_{s}(s)+Q_{L2}(s)}{\frac{K_{q2}(\frac{A_{c}R_{c}}{R_{1}}+A_{m2})}{m_{2}s^{2}+B_{2}s+K_{s2}}+(K_{c2}-\frac{V}{E}s+C_{L})}\;$$

where $K_{R}=\frac{1}{R_{1}}+\frac{1}{R_{c}}$.

The above equation clearly shows that this is a six-order transfer function. The transfer function model involves many parameters, which cannot be determined. In order to verify the correctness of the mathematical model, the method of parameter identification was used in an experiment.

**Figure 6:**
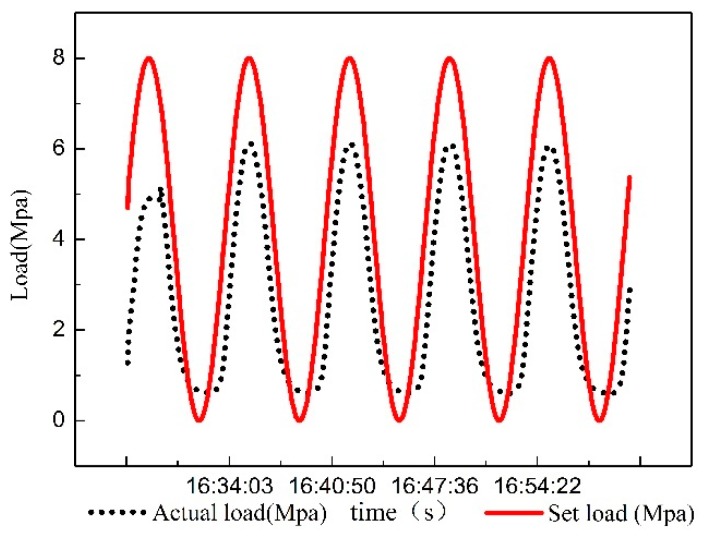
Dynamic curve diagram of real time data.

First, a sinusoidal signal with an amplitude of 4 MPa, a zero point of 4 MPa, and a cycle of 500 s is produced by the WINCC simulator. Then, the signal is converted into analog signal by PLC and is inputted to the load system. The real-time output pressure is collected through the pressure sensor. The data are shown in Figure 6.

The input and output data are collected as the data source of system identification. The parameter identification of the autoregressive moving-average model (ARMAX) is identified by the deviation elimination of the least square method [40,41].

The ARMAX model structure is

(9) $$y(t)+a_{1}y(t-1)+\ldots a_{n}y(t-n_{a})=+b_{1}u(t-n_{k})++b_{n_{b}}u(t-n_{k}-n_{b}+1)+c_{1}e(t-1)+c_{n_{c}}e(t-n_{c})+e(t)$$

A more compact way to write the difference Equation is

(10) $$A(q)y(t)=B(q)u(t-n_{k})+C(q)e(t)\;$$

where y(t) is output at time *t*, $n_{a}$ is number of poles, $n_{b}$ is number of zeroes plus 1, $n_{c}$ is number of C coefficients, $n_{k}$ is number of input samples that occurs before the input affects the output, also called the dead time in the system. $y\left(t-1\right)...y\left(t-n_{a}\right)$ are previous outputs on which the current output depends. $u\left(t-n_{k}\right)...u\left(t-n_{k}-n_{b}+1\right)$ are previous and delayed inputs on which the current output depends. $e(t-1)...e(t-n_{c})$ is the white-noise disturbance value.

The parameters $n_{a}$, $n_{b}$ and $n_{c}$ are the orders of the ARMAX model, and $n_{k}$ is the delay. *q* is the delay operator.

(11) $$\left\{ \begin{array}{l} A(q)=1+a_{1}q^{-1}+\ldots +a_{n_{a}}q^{-n_{a}}\\ B(q)=b_{1}+b_{2}q^{-1}+\ldots +b_{n_{b}}q^{-n_{b}+1}\\ C(q)=1+c_{1}q^{-1}+\ldots +c_{n_{c}}q^{-n_{c}} \end{array}\right.$$

**Figure 7:**
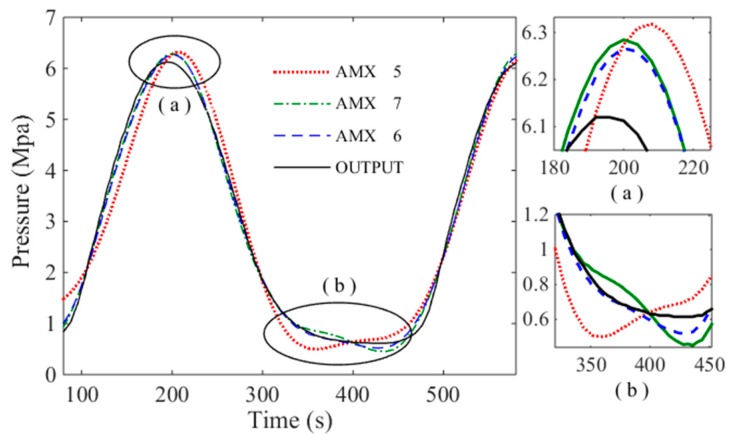
System identification contrast diagram.

The input and output data of the system shown in Figure 6 are imported into the ARMAX model. The system is identified using each order model respectively. It is found that the output of the 5–7 order model has the highest degree of fitting to the system. So, the 5/6/7-order model are chosen to draw their response curve for sinusoidal input. It can be found from Figure 7 that the best fit of the six-order system reaches 98.01%, which is more accurate than the 5th and 7th order systems. The system identification results and mathematical model correspond.

The six-order model based on the ARMAX model is shown as Equation (12):

(12) $$\left\{ \begin{array}{l} A(z)=1-2.762z^{-1}+1.786z^{-2}+1.103z^{-3}\\ \;\;-1.213z^{-4}-0.2064z^{-5}+0.2947z^{-6}\\ B(z)=-0.02795z^{-1}+0.1337z^{-2}-0.2311z^{-3}\\ \;\;+0.2577z^{-4}-0.2137z^{-5}+0.08256z^{-6}\\ C(z)=1-1.974z^{-1}+0.9825z^{-2} \end{array}\right.$$

The corresponding transfer function is:

(13) $$G(s)=\frac{-15.28s^{5}+59.92s^{4}-2659s^{3}+3.052\times 10^{4}s^{2}-6.682\times 10^{4}s+7.804\times 10^{4}}{s^{6}+19.55s^{5}+2331s^{4}+687.9s^{3}+1.396\times 10^{5}s^{2}-1.606\times 10^{6}s+1.213\times 10^{7}}$$

## 3. Simulation and Experiment

### 3.1. Simulation

The algorithm of the PID controller is an imitation of the simple and effective human operation mode [42,43]. It has the advantages of a simple algorithm and strong robustness, and it is the most widely used industrial controller. However, 10% to 20% of the industrial control problems still fail to achieve satisfactory results by using the traditional PID control strategy. These control processes often have the characteristics of nonlinearity, strong coupling and large delay. The FOPID controller was proposed by Podlubny [44,45]. It can be expressed as $PI^{\lambda }D^{\mu }$ controller. FOPID control is a new research direction in the control algorithm, which is the generalization of the traditional PID control theory. Compared with the traditional PID controller, the FOPID controller has two more adjustable parameters. The tuning range of controller parameters becomes larger. Because of this, FOPID controller is less sensitive than the classical PID controller when the parameters of the system change. Therefore, it can improve the robustness of the control system and achieve better control effect.

The basic operator of fractional calculus is Dtαa. A and t are the upper and lower bounds of the operator. α is the order of calculus. The most commonly used definitions of fractional calculus are the Riemann-Liouville (RL) definition, the Grünwald-Letnikov (GL) definition and the Caputo definition.

GL definition: Dtαaf(t)=limh→01Γ(α)hα∑k=0(t−a)/hΓ(k+α)Γ(k+1)f(t−kh).

Caputo definition: Dtαaf(t)=1Γ(m−α)∫atfm(τ)(t−τ)1+α−mdτ $m-1<\alpha <m,\begin{array}{cc} & m\in N \end{array}$

RL definition: Dtαaf(t)=1Γ(m−α)(ddt)m∫atf(τ)(t−τ)1−(m−α)dτ. $m-1<\alpha <m,\begin{array}{cc} & m\in N \end{array}$.

The definition of Gamma function is $\Gamma \left(z\right)=\int_{0}^{\infty }e^{-t}t^{z-1}dt$. The more commonly used tool for describing fractional order systems is Laplace transform. Under the definition of RL, the Laplace transform of an α−th derivative (α∈R+) of a signal *x*(*t*) at *t* = 0 is given by: $L\left\{D^{\alpha }x\left(t\right)\right\}=s^{\alpha }X\left(s\right)-\sum_{k=0}^{m-1}s^{k}{}_{0}D_{t}^{\alpha -k-1}x(t)|{}_{t=0}$. If $D_{t}^{\alpha -k-1}x(t)|{}_{t=0}=0$, *k* = 1, 2, …, *m* − 1, $L\left\{D^{\alpha }x\left(t\right)\right\}=s^{\alpha }X\left(s\right)$ can be obtained.

The general form of FOPID control is $PI^{\lambda }D^{\mu }$ controller. Its general form of the transfer function is:

(14) $$G_{c}(s)=K_{p}+\frac{K_{I}}{s^{\lambda }}+K_{D}s^{\mu }\begin{array}{ccc} & & \left(\lambda ,\mu >0\right) \end{array}$$

So, if the input signal *e*(*t*) and output signal *u*(*t*) are at *t* = 0, in the time domain, the control signal *u*(*t*) can be expressed as:

(15) $$K_{p}e\left(t\right)+K_{I}D^{-\lambda }e\left(t\right)+K_{D}D^{\mu }e\left(t\right)$$

The fractional order system is a mathematical model based on the fractional calculus equation. The realization of the controller in the application of the FOPID controller needs a reasonable method. Through the analysis of various continuous filters in the literature [46], the Oustaloup filter has good approximation effect. Therefore, the Oustaloup filter [47] is used to approximate the fractional calculus operator.

The Oustaloup method is an approximate method used to realize the fractional calculus operator in the frequency band. Its filter can be the following form.

(16) $$S^{\alpha }\approx K\prod_{k=-N}^{N}\frac{s+{\omega^{\prime }}_{k}}{s+\omega_{k}}$$

In which

(17) $$\begin{array}{l} \left\{ \begin{array}{l} {\omega^{\prime }}_{k}=\omega_{b}{\left(\frac{\omega_{h}}{\omega_{b}}\right)}^{\frac{k+N+\frac{1}{2}(1-\alpha )}{2N+1}}\\ \omega_{k}=\omega_{b}{\left(\frac{\omega_{h}}{\omega_{b}}\right)}^{\frac{k+N+\frac{1}{2}(1+\alpha )}{2N+1}} \end{array}\right.K=\omega_{h}^{\alpha } \end{array}$$

The OUSTAFOD method is to use signal filter to fit Laplace transform operator $S^{\alpha }$. α is the order of calculus; 2*N* + 1 is the order of the filter; $\left(\omega_{b},\omega_{h}\right)$ is a frequency band that needs to be fitted. The frequency range and the filter order are taken as the calculation parameters into Equation (17). ${\omega^{\prime }}_{k}$, $\omega_{k}$ and *K* are brought into Equation (16) to calculate the approximate rational transfer function. Commonly, the greater *N* is, the better the approximation effect will be. In practice, we can get a satisfactory approximation when *N* ≥ 2. So, in this paper, *N* was selected as 2. $\left(\omega_{b},\omega_{h}\right)$ are selected as (0.001,1000). As a result, the fractional calculus of the function is approximated by the output signal obtained by the original signal through such a filter.

Parameter tuning is important for the design of the controller. According to different application conditions, more and more parameter tuning methods are proposed. In the literature [48], some typical parameter-optimization methods are studied based on the Monte Carlo experiment principle. It is clear that ITAE is one of the widely applied performance indexes of SISO control system and adaptive control system. ITAE has been widely used for the parameter tuning of traditional the PID controller in the industry because of its practicability and selectivity.

ITAE is chosen as the performance index of system tuning due to the following reasons.

(1). The FOPID controller has many similarities with the traditional PID control.
(2). The loading system of the test rig is used to simulate the rolling force in the industrial rolling process.

In the simulation process, the unit step signal is used as the input signal to optimize the parameters. By using the Åström-Hägglund tuning algorithm [49], the classical PID controller is designed. Its $\left[\begin{array}{cc} K_{p} & \begin{array}{cc} K_{I} & K_{D} \end{array} \end{array}\right]$ are $\left[\begin{array}{cc} 100 & \begin{array}{cc} 99 & 6.049\times 10^{5} \end{array} \end{array}\right]$. Five parameters, including $\left[\begin{array}{cc} K_{p} & \begin{array}{cc} K_{I} & \begin{array}{cc} \begin{array}{cc} K_{D} & \mu \end{array} & \lambda \end{array} \end{array} \end{array}\right]$ of the FOPID controller, need to be designed. The desired gain margin $A_{m}$ and phase margin $\varphi_{m}$ can be used to design the fractional order PID controller [30]. From the basic definitions of gain and phase margin, the controlled system *G_p_*(*s*) and the controller *G_c_*(*s*) should satisfy the following:

(18) $$\varphi_{m}=\arg\left[G_{c}\left(j\omega_{g}\right)G_{p}\left(j\omega_{g}\right)\right]+\pi$$

(19) $$A_{m}=\frac{1}{\left|G_{c}\left(j\omega_{g}\right)G_{p}\left(j\omega_{p}\right)\right|}$$

where $\omega_{g}$ is given by $\left|G_{c}\left(j\omega_{g}\right)G_{p}\left(j\omega_{g}\right)\right|=1$, $\omega_{p}$ is given by $\arg\left[G_{c}\left(j\omega_{g}\right)G_{p}\left(j\omega_{g}\right)\right]=-\pi$. Replace *G_c_*(*s*) with Equation (14).

(20) $$K_{p}+K_{I}\frac{\cos\frac{\pi \lambda }{2}}{\omega_{p}^{\lambda }}+K_{D}\cos\left(\frac{\pi \mu }{2}\omega_{p}^{\mu }\right)=R_{mp}\;$$

(21) $$K_{p}+K_{I}\frac{\cos\frac{\pi \lambda }{2}}{\omega_{g}^{\lambda }}+K_{D}\cos\left(\frac{\pi \mu }{2}\omega_{g}^{\mu }\right)=R_{mg}\;$$

(22) $$-K_{I}\frac{\sin\frac{\pi \lambda }{2}}{\omega_{p}^{\lambda }}+K_{D}\sin\frac{\pi \mu }{2}\omega_{p}^{\mu }=I_{mp}\;$$

(23) $$-K_{I}\frac{\sin\frac{\pi \lambda }{2}}{\omega_{g}^{\lambda }}+K_{D}\sin\frac{\pi \mu }{2}\omega_{g}^{\mu }=I_{mg}\;$$

In which:

(24) $$-\frac{1}{A_{m}G_{p}\left(j\omega_{p}\right)}=R_{mp}+jI_{mp}\;\;\;\;\;\frac{-\cos\phi_{m}-j\sin\phi_{m}}{G_{p}\left(j\omega_{g}\right)}=R_{mg}+jI_{mg}\;$$

The controlled system *G_p_*(*s*), the expected loop gain and phase margin Am and *φ_m_* are known. The unknown variables *λ*, *μ*, *ω_p_* and *ω_g_* should satisfy the following constraints.

(25) $$\begin{array}{l} \left(\omega_{g}^{\lambda +\mu }-\omega_{p}^{\lambda +\mu }\right)\left(R_{mp}-R_{mg}\right)+\left(\omega_{p}^{\lambda +\mu }I_{mp}+\omega_{g}^{\lambda +\mu }I_{mg}\right)\cot\frac{\pi \mu }{2}+\\ \left(\omega_{p}^{\lambda +\mu }I_{mg}+\omega_{g}^{\lambda +\mu }I_{mp}\right)\cot\frac{\pi \lambda }{2}-\left(\cot\frac{\pi \lambda }{2}+\cot\frac{\pi \mu }{2}\right)\left(\omega_{p}^{\lambda }\omega_{g}^{\mu }I_{mp}+\omega_{g}^{\lambda }\omega_{p}^{\mu }I_{mg}\right)=0 \end{array}$$

If the parameters of $\omega_{p}$, $\omega_{g}$, λ, μ are known, $K_{I}$ $K_{P}$ $K_{D}$ can be uniquely decided as follows:

(26) $$K_{P}=\frac{\omega_{p}^{\lambda }R_{mp}-\omega_{g}^{\lambda }R_{mg}-\cot\frac{\pi \mu }{2}\left(\omega_{p}^{\lambda }I_{mp}-\omega_{g}^{\lambda }I_{mg}\right)}{\omega_{p}^{\lambda }-\omega_{g}^{\lambda }}=\frac{\omega_{g}^{\mu }R_{mp}-\omega_{p}^{\mu }R_{mg}+\cot\frac{\pi \lambda }{2}\left(\omega_{g}^{\mu }I_{mp}-\omega_{p}^{\mu }I_{mg}\right)}{\omega_{g}^{\mu }-\omega_{p}^{\mu }}\;$$

(27) $$K_{I}=\frac{\omega_{g}^{\lambda }\omega_{p}^{\lambda }\left(\omega_{g}^{\mu }I_{mp}-\omega_{p}^{\mu }I_{mg}\right)}{\sin\frac{\pi \lambda }{2}\left(\omega_{p}^{\lambda +\mu }-\omega_{g}^{\lambda +\mu }\right)}\;\;\;\;K_{D}=\frac{\omega_{P}^{\lambda }I_{mp}-\omega_{g}^{\lambda }I_{mg}}{\sin\frac{\pi \mu }{2}\left(\omega_{p}^{\lambda +\mu }-\omega_{g}^{\lambda +\mu }\right)}$$

The expected loop phase and gain margins are specified as *φ_m_* = *π*/3 and *A_m_* = 1.2. λ and μ were selected in the (0,1) scope. Take 0.001 as the step length. The corresponding $K_{I}$, $K_{P}$, $K_{D}$ and ITAE are calculated respectively. The ITAE values of different combinations are calculated and compared, and then the combination with minimum value is selected. Five parameters, including $\left[\begin{array}{cc} K_{p} & \begin{array}{cc} K_{I} & \begin{array}{cc} \begin{array}{cc} K_{D} & \mu \end{array} & \lambda \end{array} \end{array} \end{array}\right]$ of the FOPID controller, are designed to be $\left[\begin{array}{cc} 99.98 & \begin{array}{cc} 25.227 & \begin{array}{cc} \begin{array}{cc} 99.505 & 0.045 \end{array} & 0.912 \end{array} \end{array} \end{array}\right]$.

**Figure 8:**
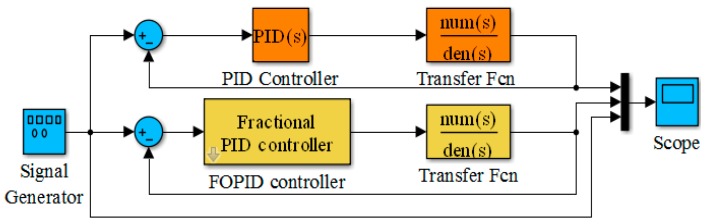
Simulation model diagram.

Simulation analysis based on MATLAB/Simulink was carried out. Because the implementation of the FOPID controller is complicated, it needs to be encapsulated first. The simulation block diagram, as shown in Figure 8, was set up to do so.

### 3.2. Experiment

**Figure 9:**
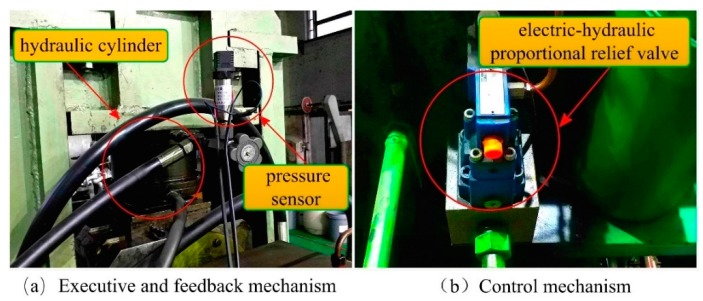
Main mechanism of Journal bearing test rig.

The main experimental equipment of the journal bearing test rig is shown in Figure 9.

The test rig uses the hydraulic cylinder to apply loads to the test bearing. The hydraulic cylinder is responsible for the simulation of rolling force and its pressure is adjusted directly by the electro-hydraulic proportional relief valve. Pressure sensors are installed next to the hydraulic cylinder to measure real-time loads are applied onto the journal bearing. A Siemens S7-300 PLC is used as controller hardware. The FOPID control algorithm is executed in the PLC controller. In the process of hydraulic load control, the monitoring picture is designed by using the WINCC configuration function. The control program runs in interrupt mode and its interrupt cycle is 100 ms. On the industrial personal computer, the set value and the process value of the working condition are collected and archived [50]. The experimental data are recorded and analyzed through the historical curve. In the experiment, a sinusoidal signal with an amplitude of 4 MPa, a zero point of 4 MPa, and a cycle of 500 s were used as input. The real-time pressure was collected through pressure sensor and the corresponding curve is drawn. The control curve is shown in Figure 13.

## 4. Results and Discussion

**Figure 10:**
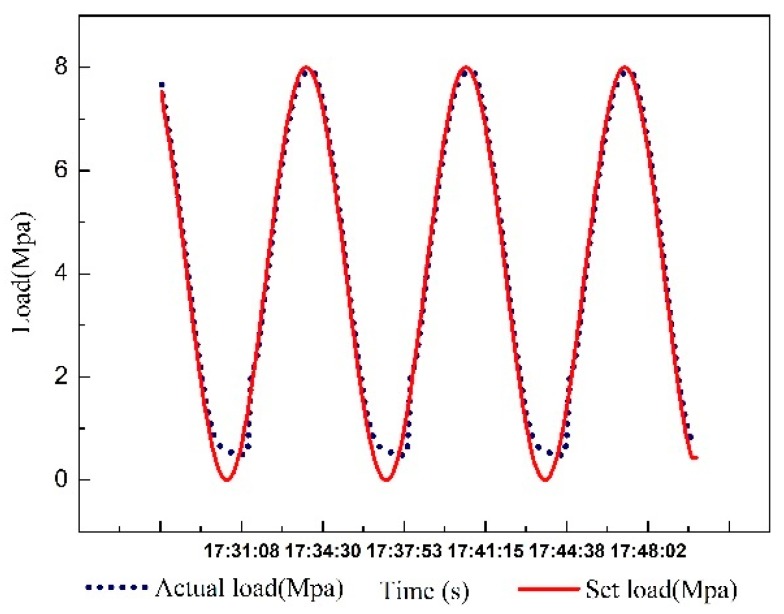
Dynamic curve diagram of real time data.

**Figure 11:**
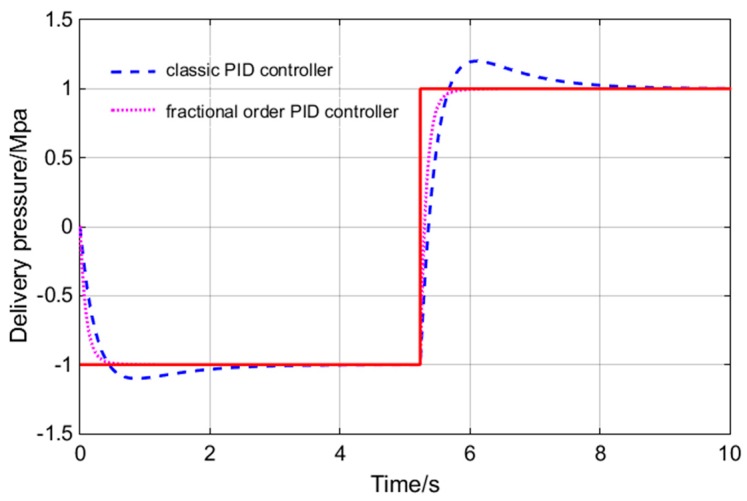
Square wave input signal tracking response.

Sine wave and square wave are used as input signal for simulation. The specific control effect is shown in Figure 10 and Figure 11.

**Figure 12:**
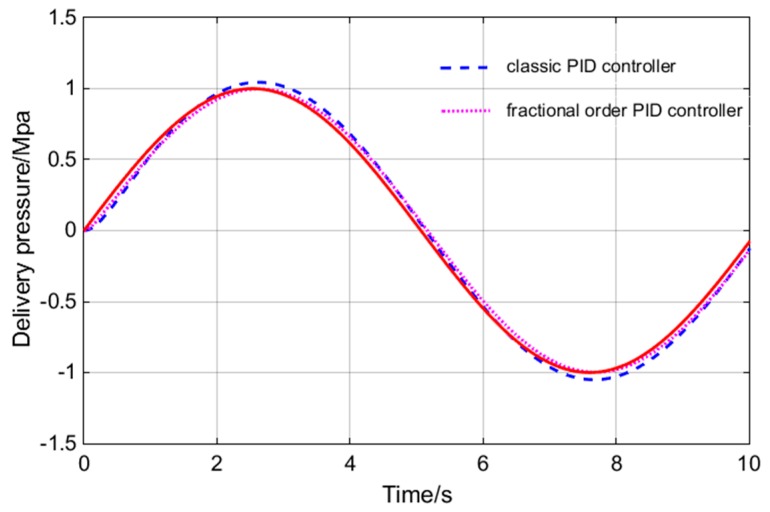
Sine input signal tracking response.

As can be seen from Figure 10 and Figure 11, the FOPID controller has faster response speed, smaller overshoot, shorter setting time, rise time and delay time than the PID controller. In practical engineering applications, overshoot is the most concerning factor of all parameters. Small overshoot can ensure that there is no oscillation of pressure values. In Figure 10, the FOPID controller has smaller overshoot. The response speed is also important for the system studied. The main function of the hydraulic loading system is to simulate constantly changing load. In order to ensure the accuracy of the experimental data, the tracking accuracy of the loading system is very important. In the Figure 11, the tracking precision of the load is up to 96%. So, the FOPID controller has obvious advantages in these two aspects. The bode diagrams for the two controllers are made respectively, as shown in Figure 12.

As can be seen from Figure 12, the FOPID controller has faster response speed and wider bandwidth than the traditional PID controller. In summary, the FOPID controller can improve the control accuracy and security of the test-rig loading system. It is more suitable for the control algorithm of the loading system.

**Figure 13:**
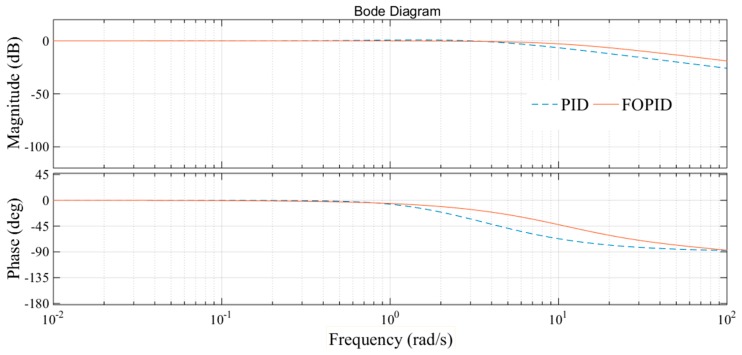
Bode plots of close loop.

As shown in Figure 13, tracking errors still occur when the pressure is low. Considering the long distance of pipeline, there may have been residual pressure in the system. The reason for this phenomenon is that the pressure in the hydraulic pipe cannot be rapidly reduced to zero in practice. This residual pressure can provide conditions for the rapid realization of the next braking. However, there is good performance in other pressure ranges. All in all, compared with Figure 6, the tracking precision of the set value is far superior. Therefore, the FOPID controller has great control effect.

## 5. Conclusions

From the above study on the performance improvement of the hydraulic system, some conclusions can be drawn.

(1). The transfer function model was obtained by system parameter identification based on the ARMAX model. The 6th order transfer function has the best fit. The mathematical model of the hydraulic loading system was set up through theoretical analysis, which is also a 6-order transfer function.
(2). In order to adapt to the hydraulic loading system, the FOPID controller and traditional PID controller were designed respectively using the tuning method based on the ITAE performance index. A series of parameters, such as $K_{d}$, $K_{I}$, $K_{p}$, λ and μ were optimized.
(3). The FOPID controller and PID controller were compared in time domain and frequency. It was found that the FOPID controller has a faster response speed and lower overshoot, which can better meet the control needs of the hydraulic loading system. In order to verify the performance of the FOPID controller on the electro-hydraulic system, experiments were carried out on the journal bearing test rig. The experimental results also prove that the FOPID controller has great control performance.

## Figures and Tables

**Table 1:** Parameters value of transfer function.

Symbol	Quantity
$K_{a}$	Proportional amplifier coefficient (A/V)
$K_{i}$	Proportion electro-magnet gain (A/V)
$B_{1e}$	Equivalent damping coefficient on armature.
$K_{1e}$	Equivalent spring stiffness (N/m)
$C_{L1}$	Leakage coefficient of pilot valve
E	Bulk modulus of elasticity of oil (N/m^2^)
$m_{1e}$	Total mass of poppet valve Spool and armature (kg)
$A_{m1}$	The action area of the liquid pressure on the end of poppet valve (m^2^)
$V_{0}$	The cavity of Pilot valve
$Q_{y1}$	The flow to cavity $V_{0}$ (m^3^/s)
$R_{0}$	Liquid resistance
$Q_{1}$	The cavity in pilot liquid bridge
$Q_{c}$	the load flow of the pilot relief valve (m^3^/s)
$P_{s1}$	Output pressure of pilot valve (Pa)
$R_{1}$	Liquid resistance
$R_{c}$	Liquid resistance
$K_{c}$	Spring stiffness (N/m)
$V_{c}$	The volume between the damping hole $R_{c}$ and the upper chamber of the main valve. (m^3^)
$p_{c}$	Pilot hydraulic bridge output pressure. (Pa)
$A_{c}$	The pressure measurement area of the main valve spool on $p_{c}$ (m^2^)
$A_{m2}$	Pressure measuring surface of system pressure (m^2^)
$Q_{y2}$	Flow of the main valve port (m^3^/s)
$Q_{2}$	Oil inlet cavity of the main valve
$P_{s}$	Output pressure of the system (Pa)
$Q_{L2}$	Load flow (m^3^/s)
$Q_{s}$	The output flow of Quantitative pump (m^3^/s)

**Table 2:** Parameters value of equation of hydraulic system.

Symbol	Quantity
$V_{1}$	Load volume of the pilot level (m^3^)
$V_{c}$	The volume between the damping hole $R_{c}$ and the upper chamber of the main valve (m^3^)
$A_{c}$	The pressure measurement area of the main valve spool on $p_{c}$ (m^2^)
$p_{c}(s)$	Pilot hydraulic bridge output pressure (Pa)
$m_{2}$	Quality of main valve spool (kg)
$x_{v2}(s)$	Displacement of the main valve spool (m)
$K_{s2}$	The spring stiffness on the main valve spool (N/m)
$p_{s}(s)$	The output pressure of the main stage of the relief valve (Pa)
$Q_{s}(s)$	The output flow of Quantitative pump (m^3^/s)
$B_{2}$	Damping ratio of main valve spool
$Q_{L2}(s)$	Load flow (m^3^/s)
V	Closed volume of pump outlet pressure zone (m^3^)
$Q_{y2}(s)$	Flow of the main valve port (m^3^/s)
$K_{q2}$	Discharge coefficient of main valve port (m^2^/s)
$K_{c2}$	Flow-pressure coefficient of main valve port (m^5^/(N·s))
$F_{L}(s)$	Disturbance power on the main valve spool (N)
$I_{\left(s\right)}$	Input current (A)
$C_{L}$	Leakage coefficient of main valve
$K_{f}$	Pressure sensor coefficient
$K_{q1}$	Flow gain of the valve port of cone valve (m^2^/s)
